# Effects of cranberry (*Vaccinum macrocarpon*) supplementation on iron status and inflammatory markers in rowers

**DOI:** 10.1186/s12970-017-0165-z

**Published:** 2017-02-28

**Authors:** Anna Skarpańska - Stejnborn, Piotr Basta, Jerzy Trzeciak, Alicja Michalska, M. Emin Kafkas, Donata Woitas - Ślubowska

**Affiliations:** 1 Department of Morphological and Health Sciences, Faculty of Physical Culture in Gorzów Wlkp. Poland, 13 Estkowskiego Str. 66 - 400, Gorzów Wlkp., Poland; 2 University School of Physical Education in Poznañ, Branch in Gorzów Wlkp., Faculty of Physical Culture, Water Sports, Gorzów Wlkp., Poland; 3 University School of Physical Education in Poznań, Branch in Gorzów Wlkp., Gorzów Wlkp., Poland; 4 Department of Coaching Education, Inonu University, School of Physical Education and Sport, Malatya, Turkey; 5 Kazimierz Wielki University Faculty of Physical Education, Health and Tourism, Bydgoszcz, Poland

**Keywords:** Cranberry, Supplementation, Strenuous exercise, Inflammation

## Abstract

**Background:**

The aim of this study was to analyze the effect of supplementation with cranberry (*Vaccinum macrocarpon*) on the levels of pro-inflammatory cytokines, hepcidin and selected markers of iron metabolism in rowers subjected to exhaustive exercise.

**Methods:**

This double-blind study included 16 members of the Polish Rowing Team. The subjects were randomly assigned to the supplemented group (*n* = 9), receiving 1200 mg of cranberry extract for 6 weeks, or to the placebo group (*n* = 7). The participants performed a 2000-m test on a rowing ergometer at the beginning and at the end of the preparatory camp. Blood samples were obtained from the antecubital vein prior to each exercise test, one minute after completing the test, and after a 24-h recovery period. The levels of hepcidin, interleukin 6 (IL-6), tumor necrosis factor alpha (TNF-alpha), ferritin, iron, soluble transferrin receptor (sTfR) and myoglobin were determined, along with total iron-binding capacity (TIBC), unbound iron-binding capacity (UIBC) and total antioxidant capacity (TAC).

**Results:**

Both prior and after the supplementation, a significant post-exercise increase in the concentration of IL-6 was observed in both groups. At the end of the study period, cranberry-supplemented athletes presented with significantly higher resting, post-exercise and post-recovery levels of TAC than the controls. However, a significant exercise-induced increase in the concentrations of TNF-alpha, myoglobin and hepcidin was observed solely in the control group.

**Conclusion:**

Supplementation with cranberry extract contributed to a significant strengthening of antioxidant potential in individuals exposed to strenuous physical exercise. However, supplementation did not exert direct effects on other analyzed parameters: inflammatory markers and indices of iron metabolism (TNF-alpha, hepcidin and myoglobin).

## Background

Normal level of iron in the body is a key determinant of health [1]. Recent studies showed that concentration of iron may be affected by many factors, inter alia by strenuous and/or long-term physical exercise. The exercise with such characteristics induces systemic inflammation, which is reflected by enhanced release of hepcidin and resultant disorders of iron metabolism. One consequence of these unfavorable changes is hypoferremia, which may eventually lead to anemia [2, 3].

Some authors tried to prevent disorders of iron metabolism, correcting its deficiency by means of dietary supplementation or intravenous injection. Reardon [4] demonstrated that iron injections may increase skeletal muscle iron content in mice. According to these authors, the elevated level of iron induced oxidative stress and reduced exercise performance. Burden [5] used a single 500 mg intravenous iron injection in Fe-deficient non-anemic runners. While an improvement of iron metabolism parameters has been demonstrated four weeks post-injection, this was not reflected by better aerobic capacity. Similar changes were also observed following oral administration of iron [6, 7]. Altogether, this evidence suggests that although supply of exogenous iron may improve its metabolic parameters, this is not reflected by expected changes in physical capacity.

Disorders of iron homeostasis are postulated to be linked to excessive generation of reactive oxygen species. Disruption of the pro-oxidant-antioxidant balance may be associated with the release of iron from active sites of proteins and resultant increase in labile iron pool (LIP). As a result, also concentration of iron ions that can take part in Fenton’s reaction increases, which exacerbates oxidative stress on the one hand, and may promote systemic inflammation and hepcidin release on the other [8].

Administration of compounds with high antioxidant potential and strong iron-chelating activity seems to be a reasonable way to normalize iron metabolism and to attenuate systemic inflammation induced by strenuous physical exercise. Such compounds are present in many fruits and vegetables, including cranberry fruits [9, 10].

American cranberry (*Vaccinium macrocarpon* Ait.), also referred to as bearberry and large cranberry (*Oxycoccus macrocarpos*), is a member of *Ericaceae* family, which had been for a long time used in traditional folk medicine, inter alia as a treatment for urinary tract infections. Many recent clinical studies and in vitro experiments confirmed that some components of cranberry fruits exert beneficial effects in the urinary tract [11, 12] and may alleviate gastrointestinal ailments associated with *Helicobacter pylori* infection [13]. Also antiviral (against reoviruses, rotaviruses and influenza viruses), antioxidant and anti-inflammatory effects of these compounds were documented [14–17].

Available evidence suggests that for the reduction of free radicals and modulation of inflammatory response are responsible polyphenolic compounds present in cranberries, such as anthocyanins, flavonols, and flavanols [10]. Bioavailability of these compounds is determined by their metabolism and absorption. The study of healthy adults conducted by McKay et al. [18] revealed a considerable individual variability in the concentration of polyphenolic compounds and their metabolites after ingestion of low-calorie cranberry juice cocktail, as well as an increase in antioxidant capacity of blood plasma. The latter has been observed for up to 8 h post-ingestion [18], which may potentially constitute an important factor in control of oxidative stress.

A review of available evidence demonstrates that previous research centered mainly around the effects of cranberry supplementation on the indices of oxidative stress and inflammatory markers in animals and humans. However, to the best of our knowledge, none of the studies analyzed if and to what extent the administration of cranberry products may modulate iron metabolism under conditions of enhanced oxidative stress and inflammation induced by strenuous physical exercise.

The aim of this study was to analyze the effects of supplementation with large cranberry extract on antioxidant capacity, selected inflammatory markers and parameters of iron metabolism in individuals exposed to strenuous physical exercise; the latter is considered as a disruptor of systemic iron turnover.

## Methods

### Study population

The study included 16 male members of the Polish Rowing Team (13 heavyweight and 3 lightweight rowers). The basic characteristics and sport classes of the athletes are presented in Table 1. Inclusion and Exclusion criteria are listed in Table 2. The study was conducted between April and June, during a 6-week training camp taking place between the preparatory and competitive phase of a yearly training cycle.

**Table 1:** Basic characteristics of the studied groups (mean ± standard deviation)

Parameters	Supplemented group (*n =* 9)	Control group (*n =* 7)
Age (years)	21.3 ± 0.82	21.3 ± 0.71
Body mass (kg)	93.6 ± 6.02	81.6 ± 9.44
Body height (cm)	192.6 ± 6.53	189.0 ± 4.62
Duration of training (years)	7.5 ± 1.5	6.9 ± 1.2

*P* = NS for all between-group comparisons

**Table 2:** Inclusion and exclusion criteria for the study participants

	Total N	Supplemented group N	Control group N
Inclusion criteria			
• Male athletes qualified to Polish Youth Rowing Team (age 19–23 years), with physician-certified permission to participate in trainings and competition	• 19	• 10	• 9
Exclusion criteria	• 3	• 1	• 2
• Medication intake (inflammatory drags, medications known to affect iron metabolism)	• 2	• 1	• 1
• Injury excluding participation in trainings	• 1	• 0	• 1

### Food intake

Throughout the entire study period, the athletes resided and took their meals exclusively at one of the Olympic Games Training Centers. Their regular menu consisted of a mixed diet, providing recommended dietary allowance (RDA) of carbohydrates, proteins, fats, and micronutrients (vitamins and minerals), as stated in the Polish Nutrition Society guidelines [19]. The athletes’ daily food, caloric, and fruit and vegetable intakes were constant throughout the study period. None of the study subjects presented with any health problems nor took any anti-inflammatory drugs, vitamins or medications that may interfere with iron metabolism. The participants were under no dietary restrictions.

### Experimental procedure

The athletes who were randomized to the supplemented group (*n* = 9) received 720-mg capsules containing highly-concentrated large cranberry extract. Each capsule contained 360 mg of cranberry extract (including 36 mg proanthocyanidins), i.e. an equivalent to 4320 mg of cranberry fruits. Aside from cranberry extract, standardized for proanthocyanidin content (10%), each capsule contained also beef-pork gelatin, filler (cellulose), anti-caking agents (magnesium salts of fatty acids, talc) and colorants (titanium dioxide, azorubine, patent blue V). Capsules were manufactured by Synoptis Pharma Sp. Z o.o., Poland, and their expiry date was specified as March 2017. The athletes ingested two capsules with cranberry extract daily (one in the morning and one in the evening) for six weeks. The subjects from the control group (*n* = 7) were given dyed gelatin placebo capsules containing “Poznańska” flour (manufactured at Polskie Zakłady Zbożowe, Krakow) twice a day for 6 weeks, at the same time and with the same dosage regimen.

### Rowing performance test

The athletes performed a controlled 2000-m time test on the first day (prior to the supplementation) and at the end of the training camp (after the supplementation). Each subject had to cover the distance on a rowing ergometer (Concept II, USA) in as short a time as possible. Because the results of both tests were taken into consideration during the selection to the championship team, the athletes were well motivated to perform both trials at a maximal effort. Each test was preceded by a 5-min individual warm-up.

### Sample treatment

Blood samples were obtained before the 2000-m test (in the morning, after an overnight fast), 1 min after completing the test, and after a 24-h recovery period. Blood samples were collected to tubes without additives and following centrifugation (10 min at 3500 rpm), the sera were stored at −80 °C until analysis. Additionally, capillary blood samples were obtained from the earlobe before and after each exercise test to assess the athletes’ lactic acid levels.

### Measurements

Serum IL-6 was measured using a commercially available enzyme-linked immunosorbent assay (ELISA; Quantikine HS, R&D Systems, Minneapolis, USA) with an assay range of 0.38-10 pg⋅ml^−1^. The intra assay and inter assay coefficients of variation (CVs) were 6.4 and 8.5%, respectively. Serum concentrations of TNF-alpha (in pg/ml) were quantified using a commercially available enzyme immunoassay (ELISA; Quantikine HS, R&D Systems, Minneapolis, USA) with an assay range of 0.5 to 32 pg⋅ml^−1^. The intra assay and inter assay CVs were 8.2 and 11.5%, respectively. Serum hepcidin was measured using a commercially available ELISA (Wuhan EIAab Science Co., China) with an assay range of 0.187-12 ng⋅ml^−1^. The intra assay and inter assay CVs were 5.2 and 7.9%, respectively. Iron concentration and TIBC were measured with the colorimetric method with chromogens (BioMaxima S.A., Poland); the results were expressed in μg/dL. Unsaturated iron-binding capacity (UIBC) was calculated from the formula: UIBC = TIBC – Fe. Myoglobin concentration was determined immunochemically, with an aid of the Myoglobin ELISA kit (BioCheck, USA); the results were expressed in ng⋅ml^−1^. The intra assay and inter assay CVs were 3.4 and 5.5%, respectively. Serum ferritin levels were determined immunochemically, with an aid of a commercially available diagnostic kit (Demeditec, Germany); the results were expressed in ng⋅ml^−1^. The intra assay and inter assay CVs were 4.6 and 6.0%, respectively. Concentrations of soluble transferrin receptor (sTfR) were determined immunochemically with a commercially available diagnostic kit (BioVendor, Czech Republic). The intra assay and inter assay CVs were 8.4 and 13.5%, respectively. Total antioxidant capacity (TAC), considered a marker of plasma antioxidant capacity, was assessed with a commercially available kit (Cayman, USA); the results were expressed as mmol⋅l^−1^. The intra assay and inter assay CVs were 3.3 and 5.7%, respectively. Concentration of lactate (La) in capillary blood was determined immediately after sampling, using a commercially available kit (Dr Lange, Germany); Lactate concentrations were expressed in mmol⋅l^−1^. If necessary, the results were adjusted for hemoconcentration, using the exercise-induced changes in hematocrit as a covariate.

### Statistical analysis

Statistical analyses were performed with STATISTICA v. 10.0 software package (StatSoft, Cracow, Poland). All parameters were compared using 2 (supplemented and placebo group) × 3 (timing of measurement) repeated measures analysis of variance (ANOVA). Normal distribution of the data was verified with Shapiro-Wilk test. Whenever significant changes were documented on ANOVA, Fisher’s *post-hoc* tests were conducted to identify the source of significant differences. Student’s unpaired *t*-test was used to compare the study groups in terms of their anthropometric characteristics. Except for the rowing time, the results of the 2000-m tests performed prior to and after the supplementation were subjected to intragroup comparisons with Student’s paired *t*-test, and for intergroup comparisons with Student’s unpaired *t*-test. The results are presented as arithmetic means ± standard deviations (SD). The threshold of statistical significance for all tests was set at *p* < 0.05.

## Results

The subjects from the supplemented group and the controls did not differ significantly in terms of their mean age, body height, body weight, and years of training (Table 1).

No significant intergroup differences were found in mean power output and total row time during the 2000-m test performed at the beginning of the training camp. Furthermore, no significant differences in the pre- and post-test blood lactate levels were documented when the results for Trial I were compared to those of Trial II (Table 3).

**Table 3:** Changes in 2000 m rowing ergometer performance before and after supplementation

Parameters	Supplemented group (*n =* 9)		Control group (*n =* 7)	
	Before	After	Before	After
Power (watt)	452 ± 15.6	457 ± 12.5	406 ± 36.6	417 ± 39.5
(W x kg^−1^)	4.84 ± 0.25	4.88 ± 0.28	4.99 ± 0.28	5.03 ± 0.43
LA_min_ (mmol x L^−1^)^a^	1.8 ± 0.23	1.8 ± 0.19	1.8 ± 0.26	1.9 ± 0.18
LA_max_ (mmol x L^−1^)^a^	14.2 ± 2.91	15.0 ± 2.09	15.6 ± 2.14	16.8 ± 1.44
Time (s)	366.7 ± 4.39	365.3 ± 3.24	380.8 ± 11.34	377.2 ± 11.86

Values represent the mean ± standard deviation. There were no significant differences after supplementation relative to before supplementation (*P* < 0.05)

^a^ *LA* lactate acid

**Fig. 1:**
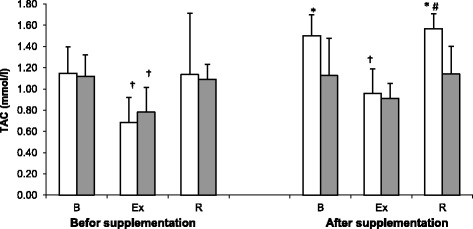
Total antioxidant capacity (TAC) levels during exercise tests performed before and after the supplementation (mean ± *SD*). *Note*. □ - SUPL = supplemented group;  − PLA = placebo group; B = baseline; Ex = immediately after the exercise; R = after a 1-day recovery; * - significantly different compared to PLA group; † − significantly different compared to baseline level; # -significantly different compared to post-exercise level

Values of total antioxidant capacity (TAC) determined at Trial I and II are shown on Fig. 1. This parameter turned out to be modulated both by physical exercise (*p* < 0.001) and cranberry supplementation. Prior to the supplementation (Trial I), both groups of athletes showed a significant post-exercise decrease in serum TAC. At trial II, cranberry-supplemented athletes presented with significantly higher resting, post-exercise and post-recovery levels of TAC than the controls. These were the athletes from the supplemented group, however, who showed a significant post-exercise decrease in serum TAC at Trial II.

**Fig. 2:**
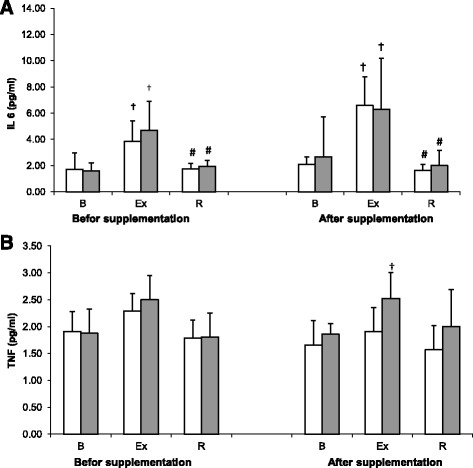
Changes interleukin 6 (**a**) and tumor necrosis factor alpha (**b**) levels during exercise tests performed before and after the supplementation (mean ± *SD*). *Note*. IL-6 = interleukin 6; TNF α = tumor necrosis factor alpha; □ − SUPL = supplemented group;  − PLA = placebo group; B = baseline; Ex = immediately after the exercise; R = after a 1-day recovery; † − significantly different compared to baseline level; # -significantly different compared to post-exercise level

Changes in serum levels of IL-6 observed prior to and after cranberry supplementation are shown on Fig. 2a. A significant effect of physical exercise on IL-6 concentration (main effect, *p* < 0.001) has been documented on ANOVA. At both Trial I and II, a significant post-exercise increase in the concentration of this cytokine was observed in either group, with subsequent normalization at the pre-exercise level following a 24-h recovery. Supplementation with cranberry extract did not exert a significant effect on IL-6 concentration (main effect, *p* = 0.051).

Exercise-induced changes in TNF-alpha levels are presented on Fig. 2b. Strenuous physical exercise induced statistically significant changes in this parameter, as confirmed on ANOVA (main effect, *p* < 0.001). While no statistically significant differences between pre-exercise, post-exercise and post-recovery values of this parameter were found in both the cranberry-supplemented athletes and the controls at Trial I, athletes from the latter group showed a significant exercise-induced increase in serum concentration of TNF-alpha at Trial II. Based on the results of ANOVA, cranberry supplementation did not exert a statistically significant effect on TNF-alpha levels (main effect, *p* = 0.118).

**Fig. 3:**
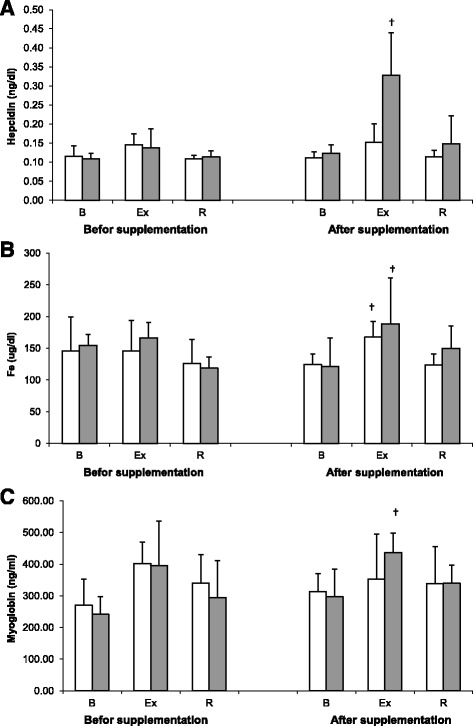
Changes in serum levels of hepcidin (**a**), iron (**b**), and myoglobin (**c**) levels during exercise tests performed before and after the supplementation (mean ± *SD*). *Note*. □ - SUPL = supplemented group;  − PLA = placebo group; B = baseline; Ex = immediately after the exercise; R = after a 1-day recovery; † − significantly different compared to baseline level

At trial II, a significant post-exercise increase in hepcidin level was observed in the controls but not in the cranberry-supplemented athletes. However, no significant effect of cranberry supplementation on this parameter was demonstrated on ANOVA (main effect, *p* = 0.177; Fig. 3a).

Supplementation with cranberry extract did not exert a significant effect on serum iron (main effect, *p* = 0.516). However, a significant post-exercise increase in serum concentrations of iron was documented in both the cranberry-supplemented athletes and the controls at Trial II (Fig. 3b).

Serum concentration of myoglobin was modulated solely by physical exercise (main effect, *p* < 0.001). While no significant exercise-induced changes in this parameter were observed in both groups at Trial I, a significant increase in serum concentration of myoglobin was documented in the controls at Trial II (Fig. 3c).

**Fig. 4:**
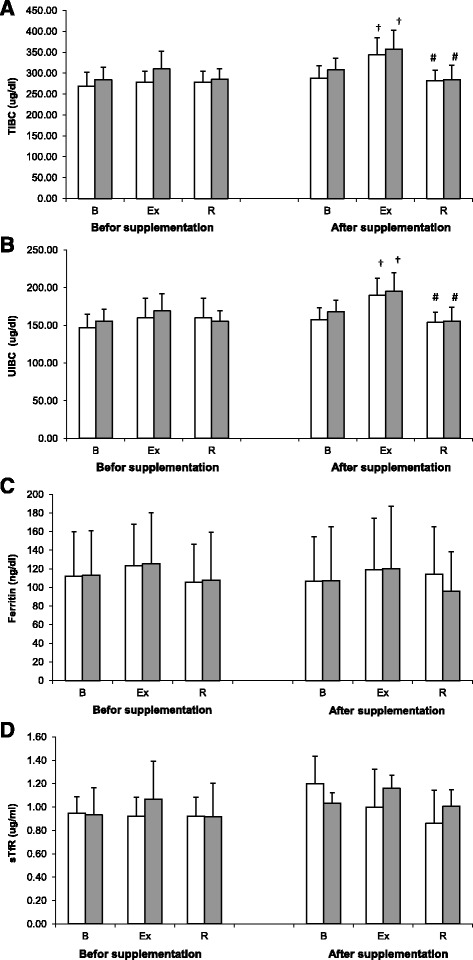
Changes TIBC (**a**), UIBC (**b**), ferritin (**c**) and sTfR (**d**) levels during exercise tests performed before and after the supplementation (mean ± *SD*). *Note*. TIBC = total iron-binding capacity; UIBC = unsaturated iron-binding capacity; sTfR = soluble transferrin receptor - □ SUPL = supplemented group;  − PLA = placebo group; B = baseline; Ex = immediately after the exercise; R = after a 1-day recovery; † − significantly different compared to baseline level; # -significantly different compared to post-exercise level

Irrespective of the group, statistically significant exercise-induced changes in TIBC (Fig. 4a) and UIBC (Fig. 4b) were observed at Trial II, but not at Trial I. Both parameters increased in response to physical exercise and then returned to their pre-exercise levels following the recovery period. Based on the results of ANOVA, cranberry supplementation did not exert a significant effect on any of these parameters.

The data on sTfR and transferrin levels are presented on Fig. 4 (c and d). Neither cranberry supplementation nor physical exercise exerted statistically significant effects on these parameters.

## Discussion

The aim of our study was to analyze the effects of large cranberry intake on antioxidant capacity, selected parameters of iron metabolism and inflammatory markers in athletes exposed to strenuous physical exercise. The study showed that physical exercise contributed to a significant decrease in serum TAC (Fig. 1). Similar findings were previously reported by Finaudi [20] (2006) and Margonis [21], according to whom pre-competitive and competitive phases of a yearly training cycle are associated with enhanced oxidative stress and a decrease in antioxidant capacity. Post-exercise decrease in TAC may result in accumulation of inadequately deactivated free radicals that may initiate peroxidation of polyunsaturated fatty acids of erythrocyte membranes [22]. Fiorani [23] demonstrated that human erythrocytes can uptake flavonoids from extracellular milieu via a passive diffusion mechanism, becoming a natural reservoir of these compounds. While most flavonoids (up to 85%) are found in the cytosolic fraction, some are bound to plasma membranes. Arora [24] and Terao [25] showed that flavonoids, similar to cholesterol and alpha-tocopherol, are localized near the surface of the membrane, between phospholipid bilayer and aqueous phase. Such location of flavonoids is crucial for stabilization of the membrane, making it less prone to oxidation due to a decrease in its fluidity [26].

Our findings imply that administration of cranberry extract may stimulate an increase in serum TAC (Fig. 1). Compared to the controls, cranberry-supplemented athletes presented with significantly higher levels of TAC at rest and following a 24-h recovery. Also other authors observed an increase in antioxidant potential after cranberry supplementation [27, 28]. According to Basu [29], administration of cranberry juice (480 ml/day for 8 weeks) contributed to a significant increase in plasma antioxidant capacity, as well as to a decrease in oxidized LDL and malondialdehyde levels in individuals diagnosed with metabolic syndrome. Similar results were also documented in a group of healthy men who consumed cranberry juice (7 ml/kg body weight per day) for 14 days [30]. Celik [31] showed that antioxidant capacity of cranberry fruits is greater when they accumulate more anthocyanins. Probably, this association can be explained by previously documented ability of anthocyanins to bind toxic iron ions [32].

Physical exercise, especially strenuous, induces an array of unfavorable changes, such as a shift in the pro-oxidant-antioxidant balance toward oxidation, hyperthermia, metabolic acidosis, hypoglycemia and hemoconcentration. All of them may contribute to a decrease in the osmotic resistance of erythrocytes and make them more susceptible to hemolysis [33]. Enhanced hemolysis may in turn lead to a dramatic increase in circulating pool of redox-active iron. Fenton’s reaction, a consequence of increased availability of Fe, constitutes the principal mechanism of iron toxicity. High reactivity and low specificity of ^•^OH substantially hinder protection of cell components and body fluids against the toxic effects of iron. After being released from cells, iron may be bound by ferritin or low-molecular-weight cytoplasmic ligands, forming the so-called labile iron pool (LIP). As a crossroad of cell iron metabolism, cytoplasmic LIP undergoes dynamic changes. LIP is built of iron that has been delivered to the cell or recovered from degraded intracellular Fe-binding proteins, and serves as a source of this metal for synthesis of heme and iron-sulfur clusters. Kvam [34] demonstrated that iron released during enzymatic degradation of heme contributes to a rapid increase in the LIP; according to these authors, this may result in a transient (2- to 3-h) increase in the susceptibility of erythrocytes to UVA-induced oxidative stress.

Both cranberry supplemented athletes and controls participating in our study showed a significant post-exercise increase in serum iron (Fig. 3b), TIBC and UIBC at Trial II (Fig. 4a and b). Elevated serum levels of iron not only may trigger free radical-mediated processes, but also activate immune system and induce inflammation as a form of an early protective response [35]. Available evidence suggests that some constituents of cranberry fruits may act as immunostimulants, mitigating the inflammation [9].

Supplementation with cranberry extract did not alter significantly the levels of inflammatory markers, IL-6 and TNF-alpha, in our athletes. Statistically significant changes in these parameters were observed after exposure of the study subjects to strenuous physical exercise (Fig. 2 a and b). The exercise-induced increase in IL-6 level was observed irrespective of the group, at both Trial I and II. Then, following the 24-h recovery, IL-6 returned to its baseline level (Fig. 2a). While serum levels of TNF-alpha in athletes from both groups did not undergo significant changes at Trial I, a significant post-exercise increase in this parameter was observed in the controls at Trial II (Fig. 2b). According to Rämson [36], high-volume training stimulated an increase in plasma TNF-alpha concentration in male rowers. Similar results were also obtained in a study of mice subjected to a high-intensity physical training [37]. These findings suggest that a post-exercise increase in TNF-alpha level may be a useful marker of exercise load accumulation and predict the onset of possible overreaching/overtraining in athletes. Noticeably, we did not observe a post-exercise increase in the serum concentration of TNF-alpha in cranberry-supplemented athletes, although they were exposed to exactly the same exercise regimen as the controls.

Anthocyanins, which are inter alia present in cranberry fruits, exert anti-inflammatory effect due to their ability to inhibit principal enzymes activated during the course of inflammation, i.e. cyclooxygenase 2 (COX-2) and lipoxygenase. Due to such properties, anthocyanins may block synthesis of inflammatory mediators, including prostaglandin E. A study using Caco-2/15 cells demonstrated that cranberry extract may prevent iron/ascorbate-mediated lipid peroxidation and counteract lipopolysaccharide-mediated inflammation, as evidenced by the decrease in pro-inflammatory cytokines: TNF-alpha, IL-6, cyclooxygenase-2 and prostaglandin E2 [15]. However, in a study of human subjects with metabolic syndrome, 60-day supplementation with cranberry juice did not cause significant changes in the levels of pro-inflammatory cytokines: TNF-alpha, IL-1 and IL-6, despite an improvement of some cardiovascular risk factors [38].

Immediately after the exercise, our rowers from the control group presented not only with the increase in TNF-alpha concentrations, but also with elevated levels of hepcidin (Fig. 3a) and myoglobin (Fig. 3c). Elevated level of myoglobin after exercise is a marker of muscle injury, whereas the increase in hepcidin is typically associated with disruption of iron metabolism [2]. Previous studies showed that the release of hepcidin is modulated by the availability of iron [39], inflammation [3], hypoxia [40] and changes in erythropoietic activity [41]. All these factors are also associated with strenuous physical exercise. Burden [5] showed that availability of iron is a key regulator of hepcidin level in elite athletes. In their study, a single intravenous injection of iron stimulated an increase in hepcidin level in iron-deficient non-anemic runners; this effect was independent of changes in IL-6 concentration. This suggests that the lack of post-exercise changes in TNF-alpha, myoglobin and hepcidin levels in supplemented athletes might reflect indirect beneficial effects of cranberry extract components: stimulation of antioxidant capacity and reduction of iron availability due to chelation [42].

Neither strenuous physical exercise no cranberry intake exerted a significant effect on the remaining parameters of iron metabolism in our rowers, i.e. sTfR and ferritin levels (Fig. 4c and d). This observation is consistent with the results published by other authors [5, 43–45].

## Conclusion

Supplementation with cranberry extract contributed to a significant strengthening of antioxidant potential in individuals exposed to strenuous physical exercise. However, supplementation did not exert direct effects on other analyzed parameters: inflammatory markers and indices of iron metabolism (TNF-alpha, hepcidin and myoglobin). The lack of exercise-induced changes in inflammatory markers and parameters of iron metabolism seems to be indirectly linked to the enhancement of antioxidant potential. However, verification of this hypothesis requires further research on the mechanisms through which various components of cranberry extract, especially anthocyanins, may interfere with iron metabolic parameters. Furthermore, there is a need for specific guidelines regarding form, timing, frequency and dosage of cranberry supplements.

## Abbreviations

ANOVA: Aanalysis of variance
COX: Cyclooxygenase
CVs: Coefficients of variation
Fe: Serum iron
IL-6: Interleukin
LA: Lactic acid
LDL: Low density lipoprotein
LIP: Labile iron pool
MCV: Mean corpuscular volume
OH: Hydroxyl radicals
RDA: Recommended dietary allowance
SD: Standard deviations
sTfR: Soluble transferrin receptor
TAC: Total antioxidant capacity
TIBC: Total iron binding capacity
TNF-α: Tumor necrosis factor alpha
UIBC: Unsaturated iron-binding capacity
UVA: Ultraviolet
